# Primary care-based models of care for osteoarthritis: a scoping review protocol

**DOI:** 10.12688/hrbopenres.13260.2

**Published:** 2021-10-15

**Authors:** Joice Cunningham, Frank Doyle, Jennifer M. Ryan, Barbara Clyne, Cathal Cadogan, Elizabeth Cottrell, Susan M. Smith, Helen P. French

**Affiliations:** 1 School of Physiotherapy, Royal College of Surgeons in Ireland, RCSI, University of Medicine and Health Sciences, Dublin, Ireland; 2 Department of Health Psychology, RCSI, University of Medicine and Health Sciences, Dublin, Ireland; 3 Public Health and Epidemiology, RCSI, University of Medicine and Health Sciences, Dublin, Ireland; 4 HRB Centre for Primary Care Research, Department of General Practice, RCSI, University of Medicine and Health Sciences, Dublin, Ireland; 5 School of Pharmacy and Pharmaceutical Sciences, Trinity College Dublin, the University of Dublin, Dublin, Ireland; 6 School of Medicine, Keele University, Keele, UK

**Keywords:** Osteoarthritis, Model of Care, Primary Care, Scoping Review, Evidence-based Guidelines

## Abstract

**Background: **The burden of osteoarthritis (OA) to individuals and health systems is substantial and is expected to increase due to population ageing and rising prevalence of obesity and multimorbidity. Primary care-based models of care (MoCs) are being increasingly developed in response to this growing burden. However, these MoCs have yet to be formally reviewed. A MoC can be defined as an ‘evidence-informed strategy, framework or pathway that outlines the optimal manner in which condition-specific care should be delivered to consumers within a local health system’.

**Objective:** To identify and describe the available research regarding the extent, nature and characteristics of MoCs for OA that have been developed or evaluated in primary care.

**Methods: **A scoping review will be conducted in accordance with the Arksey and O’Malley scoping review framework and the PRISMA-ScR guidelines. Systematic literature searches of MEDLINE, EMBASE, CINAHL, PsychINFO, Web of Science and LILACs will be conducted from 2010 to present, aligning with publication dates of recent clinical guidelines. A structured iterative search of grey literature will be conducted. Full-text original quantitative or mixed method studies which describe the development or evaluation of MoCs for OA in primary care will be considered. Data will be charted and synthesised and a narrative synthesis will be conducted.

**Conclusions: **This scoping review will provide a broad overview regarding the extent, nature and characteristics of the available literature on primary care based MoCs for OA. Findings will be used to identify gaps in the current evidence to identify areas for future research.

## Introduction

Osteoarthritis (OA) is the most common joint disease in the world ^1^ and is characterised by abnormal joint tissue metabolism, cartilage degradation, bone remodelling, osteophyte formation, joint inflammation, and loss of normal joint function ^2^. It currently ranks as the 15 ^th^ highest contributor to disability globally ^3^, affecting more than 500 million people worldwide ^4^. After diabetes and dementia, it is the third fastest growing cause of years lived with disability ^3^, with the number of people living with OA globally rising by 48% from 1990 to 2019 ^4^. Primarily diagnosed and managed in primary care settings by general practitioners (GPs) ^5^, it is one of the most frequent reasons for consultations with older adults in primary care settings ^6^. OA places a substantial burden on the individual, the community and the health system ^7^. At the individual level, OA contributes to considerable physical and psychosocial burden, frequently resulting in significant pain, physical disability, sleep interruption, poorer quality-of-life, depression, impaired work and social participation, and higher healthcare costs ^8, 9^. Commonly co-morbid with other chronic health conditions such as hypertension, heart disease and diabetes ^10^, OA negatively impacts upon the morbidity and mortality associated with these conditions ^11^. In the absence of a cure for OA, treatment should focus on pain management and improving function and health-related quality of life ^12^. Despite numerous international guidelines endorsing exercise, weight management and education as first-line conservative treatments for OA ^13– 15^, a substantial evidence-practice gap persists ^16– 19^. Currently up to 50% of patients who undergo joint replacement surgery may not have received structured education and exercise prior to their operation ^18, 20^, despite an estimation that up to a quarter of knee arthroplasty surgeries could be avoided through optimal use of non-surgical treatments ^21– 23^. Furthermore, there is evidence that many individuals with knee OA have inadequately managed pain ^24^, underscoring the need for management strategies to align with international evidence-based guidelines.

A range of factors drive the persistent evidence-practice gap in OA management including health systems and policy, socio-economic factors, delivery systems, infrastructure, volume and training of health care professionals and consumer participation and engagement ^25^. The term Model of Care (MoC) is used to describe clinical service delivery initiatives to consumers, and is being increasingly applied to musculoskeletal conditions ^26^. A MoC can be defined as an ‘evidence-informed strategy, framework or pathway that outlines the optimal manner in which condition specific care should be delivered to consumers within a local health system’ ^27^. The aim of a MoC is to explicitly operationalise evidence-based guidelines and therefore support implementation by clinical teams in their local health systems ^26^. While a growing number of primary care based MoCs for the management of OA are being developed internationally ^12, 28^, they have yet to be formally reviewed. There is a need to synthesise this body of work to identify and establish the evidence for MoCs for OA in primary care. Understanding the optimal MoCs for OA is vital to inform the design of services to optimise care. Therefore, the aim of this scoping review is to identify and describe the available evidence regarding the extent, nature, characteristics and impact of MoCs for OA management that have been developed and/or evaluated in primary care.

## Methods

### Study design

A scoping review was deemed the most suitable review methodological approach, given the broad and heterogeneous nature of the research questions to be addressed. This review may act as a valuable platform to identify topics for more focused systematic reviews and/or meta-analyses. The scoping review framework proposed by Arksey and O’Malley ^29^ will be employed. This framework suggests five steps for a rigorous scoping review: (1) identifying the research questions; (2) searching for relevant studies; (3) selecting studies; (4) charting the data, and; (5) collating, summarising, analysing and presenting the results. This framework highlights the need for scoping reviews to be an iterative process, based on initial searches, producing best results, and expert discussion ^30^. The Preferred Reporting Items for Systematic reviews and Meta-Analyses (PRISMA) extension for Scoping Reviews (PRISMA-ScR) checklist will be used to guide the reporting of this review ^31^. This protocol is reported in line with the PRISMA-P checklist ^32^.

### Stage 1: Identifying the research questions

The main research question using the Population-Concept-Context (PCC) framework, recommended by the Joanna Briggs Institute (JBI) for scoping reviews, is: What is the available evidence regarding the extent, nature and characteristics of MoCs (concept) for OA management (population) that have been developed and/or evaluated in primary care (context)? Sub-questions underpinning this overarching question include the following:

1. How were the MoCs developed and defined (including underlying frameworks, service user involvement, research designs and methods employed)?
2. What are the components of the MoCs (including prioritisation of these components if specified)?
3. What outcome measures are reported and/or recommended in studies of MoCs?
4. What are the findings reported in studies evaluating the MoCs (e.g., effectiveness, acceptability, barriers or facilitators to implementation)?

***Eligibility criteria.*** The PCC framework will be used to guide study selection and to align the eligibility criteria with the research questions ^33^. We will include original research which investigate MoCs (concept) that have been developed and/or evaluated for people with OA (population) presenting to primary care (context). Full-text quantitative or mixed-methods studies published from 2010 onwards to align with publication dates of recent guidelines will be included. No language restrictions will be applied to peer reviewed publications. However, non-peer reviewed articles will be limited to the English language, due to time and resources required for translation and interpretation. In the case of abstracts or protocols being retrieved, attempts will be made to access full-texts by contacting the authors. Inclusion and exclusion criteria are summarised in Table 1.

**Table 1.** Eligibility criteria of study selection.

Inclusion Criteria	Exclusion Criteria
Quantitative or mixed-methods studies. Quantitative studies may include both experimental (e.g., randomised trials, non- randomised trials) and observational (e.g., cohort, cross-sectional) study designs	Invalid study type: stand-alone qualitative research, case series (<10 participants) and individual case reports, opinion/narrative/ discussion/editorial or review papers
People with OA must represent ≥ 50% of the study sample	Research published only as an abstract or protocol
Full-text peer reviewed articles (no language restriction)	Research published prior to 2010
Full-text non-peer-reviewed articles (English language only)	Clinical guidelines with no implementation element
Research published from 2010 onwards to align with publication dates of recent guidelines	MOCs that focus solely on adjunct therapies and do not include self-management, education, exercise and/or dietary intervention
MoCs must be based on internationally recognised evidence- based guidelines for OA	

***Population.*** In the context of this review, MoCs must be designed for community dwelling adults (≥18 years) with diagnosed OA. OA can involve any joint, however, preferentially affects joints in the hands, knees, hips and spine ^34^. For the purpose of this review, inclusion will not be restricted to any specific joints. Diagnosis may be based on radiographic criteria, clinical features or combination criteria ^35^, and will be determined by the original research articles. Included studies are not required to specify the diagnostic criteria for OA. In studies including populations with other forms of arthritis or chronic diseases, individuals with OA must represent at least 50% or more of the study sample.

***Concept.*** The concept explored by this scoping review is primary care based MoCs for OA that have been developed and/or evaluated. The explicit use of the term ‘MoC’ is not required for inclusion. For the purpose of this review, inclusion will be based on a MoC being defined as a ‘person-centered and principle-based guide that describes evidence-informed, best practice care for OA, including what care should be provided and how it should be delivered in primary care at a regional or national level’ ^36^. A MoC is distinct from a clinical practice guideline, in that the fundamental purpose of a MoC is to operationalise ‘evidence into practice’ rather than to grade evidence and/or develop specific clinical practice recommendations ^25^, e.g. by including an implementation plan. In this way a MoC complements a clinical practice guideline by describing how evidence-based guidelines can be implemented as a sector-wide model of service delivery by clinicians, consumers and health systems across the disease continuum, while considering practicalities of the local environment. A MoC should be designed as an alternative to ‘usual care’. Therefore, studies comparing a MoC to ‘usual care’ will be included, while head-to-head comparisons of different MoCs will not. The MoC must include at least one of the core recommended treatments for OA in line with international evidence-based guidelines developed by expert consensus ^13– 15^ namely self-management, education, exercise and/ or dietary weight management, to be considered for inclusion in this review.

***Context.*** This review will consider information sources including original research, which describe the process of development of a MoC or evaluate the effectiveness of a MoC for OA in primary care settings. The setting for initiation and delivery of the MoC must be in primary care involving primary care physicians, nurse practitioners and/ or other primary healthcare professionals. The MoC may include pathways for referring patients to secondary care. However, MoCs which are initiated and delivered in secondary care or other ambulatory specialty settings will be excluded. The context will not be limited to specific geographic location. The terms primary health care, primary care, general practice and family medicine are often used interchangeably. Short descriptions of each as defined by the World Health Organisation (WHO) can be found in the online supplementary material (see *Extended data* ^37^).

### Stage 2: Identifying relevant studies

A comprehensive search strategy aimed to identify relevant literature from a broad range of sources including electronic databases, reference lists and grey literature will be developed in conjunction with a medical librarian. For the purpose of this scoping review, we will follow the three-step search strategy process recommended by the JBI ^33^. Search strategies and search terms for each included database will be devised. The following electronic databases will be searched: Ovid MEDLINE, EMBASE, and CINAHL via EBSCOhost, PsychINFO, Web of Science and LILACs. The first step will involve a limited preliminary search of Ovid MEDLINE to identify articles relevant to the topic area. Key words and index terms will be identified from the title and abstract of relevant articles and will be used to inform the final search strategy. The search strategy for Ovid MEDLINE is available as extended data ^37^. This will be modified for each database. A second comprehensive search using all identified keywords and index terms will subsequently be undertaken across all included electronic databases. The third step will involve searching for additional studies using the reference lists of identified articles. This will involve backward reference searching i.e., checking the reference lists of all identified reports and articles for additional studies, and grey literature searching. We will search a variety of grey literature sources including databases that specialise in grey literature, controlled trial registers, international government organizations and agencies, relevant scientific research groups and doctoral dissertations (see *Extended data* ^37^).

### Stage 3: Selecting studies

After the search is completed, the citations of the final included studies will be imported to EndNote X9 and duplicates removed. The study selection process will be implemented over three stages. The first step will involve two independent review authors (JC, HPF) screening titles for inclusion in the review as specified in the eligibility criteria in the first stage. A third review authors (FD) will act as an arbitrator in the event of any disagreements. In the second stage of the selection process, the same two review authors will apply the inclusion criteria to all abstracts. Studies identified through the first two steps will be uploaded in full to EndNote library, followed by a review of full-text articles. This will be conducted by the two review authors (JC, HPF) to determine their inclusion based on the study inclusion criteria. 

### Stage 4: Charting the data

This stage will involve extraction of all relevant data from each included study, to inform the scoping review objectives and questions. Data charting will be conducted via a standardised form created using Microsoft Excel software developed from the JBI data extraction tool ^33^. Two review authors will independently pilot the form on a random sample of included reports to test its applicability and it will be revised accordingly. Data will be independently charted by one researcher (JC) and cross-checked against original articles by a second researcher (HPF) to ensure the validity of extracted data. A descriptive summary of the results will be performed by charting the data. The data charted will include specific details about the population, concept, context, study methods and key findings significant to the scoping review questions. Authors of papers will be contacted to request missing or additional data, where required.

***Critical assessment for level of evidence.*** Critical appraisal of studies included in a scoping review, while not consistently performed, is encouraged. The appropriate JBI Critical Appraisal tool will be used to assess the methodological quality of included studies depending on the study design ^38^, e.g. the JBI critical appraisal checklist for randomised controlled trials and cohort studies. This will be used to inform conclusions and recommendations from the scoping review. Two review authors (JC) and (HPF) will independently appraise the included studies, with a third review author (JR), available as an arbitrator in the event of any disagreements.

### Stage 5: Collating, summarising, analysing and presenting the results

The scoping review results will be collated and summarised according to the review questions and eligibility criteria (PCC framework). Results of the literature search and study screening process will be presented in a PRISMA-ScR flow diagram. Charted data will be synthesised quantitatively and presented in tabular form which will be developed and refined throughout the data extraction. A narrative summary will accompany the tabulated results and describe how the results relate to MoCs for OA in primary care. Suggestions for future research based on the study findings will also be summarised.

## Discussion

Despite the already considerable and escalating societal, economic and personal burden of OA, it is frequently overlooked in national and global strategic plans for chronic disease management, with prioritisation given to other non-communicable diseases such as heart disease, diabetes, chronic obstructive pulmonary disease and mental health problems ^4^. With increasing healthcare utilisation and costs, the global health and socioeconomic impact of OA is currently unsustainable and constitutes a major worldwide challenge ^39, 40^. ‘Care as usual’ traditional OA management approaches often result in varying treatments, evidence-practice gaps and fragmented and delayed treatment due to waiting lists and overdemand. Therefore, a ‘paradigm shift’ in OA management is urgently required to promote evidence-informed OA management which addresses the underutilisation of core recommended treatments and over-reliance on pharmacological agents and surgery ^4^. This scoping review will synthesise the existing research on primary care based MoCs for people with OA and provide a narrative synthesis of the data based on our review questions. The results will provide an overview of the characteristics, delivery, outcomes, and outcome measures embedded in the MoCs to inform the existing approach and effort to develop new MoCs for OA in primary care. Furthermore, the results will guide future research towards developing, implementing, and evaluating appropriate MoCs tailored to individual healthcare systems.

## Dissemination of findings

On completion of the analysis, this review will be submitted for publication in a peer-reviewed journal.

## Study status

At the time of publication of this protocol, database searches have been completed and study selection is underway. 

## Data availability

### Underlying data

No underlying data are associated with this article.

### Extended data

Open Science Framework: Primary Care-Based Models of Care for Osteoarthritis; a scoping review protocol. https://doi.org/10.17605/OSF.IO/H47WJ ^37^.

This project contains the following extended data:

- Ovid MEDLINE Search Strategy
- PRISMA-P-checklist
- World Health Organization terminology relating to Primary Care
- Grey Literature Search Strategy

### Reporting guidelines

Open Science Framework: PRISMA-P checklist ^32^ for Primary care-based models of care for osteoarthritis: a scoping review protocol. https://doi.org/10.17605/OSF.IO/H47WJ ^37^.

Data are available under the terms of the Creative Commons Zero "No rights reserved" data waiver (CC0 1.0 Public domain dedication).
