# Supplementary material for: Primary care-based models of care for osteoarthritis: a scoping review protocol
Source: HRB Open Res. 2021 Oct 15;4:48. Originally published 2021 May 10. [Version 2] doi: 10.12688/hrbopenres.13260.2 (PMC8264805; doi:10.12688/hrbopenres.13260.2)
Supplement: Peer review history

# Peer review: Reviewer response for version 1

**DOI:** 10.21956/hrbopenres.14431.r29463

**Published:** 2021-07-07

**Authors:** J. Haxby Abbott

**Affiliations:** 1 Center for Musculoskeletal Outcomes Research, Department of Surgical Sciences, University of Otago, Dunedin, New Zealand

- This is a useful and worthwhile topic, well defined using the JBI PCC framework.
- The methods are well described and complete in relation to the PRISMA-ScR and JBI guideline. 
- Item 11 of PRISMA-ScR "data items" is partly detailed, but not explicitly or extensively covered.
- The writing is clear and succinct. 

Is the study design appropriate for the research question?

Yes

Is the rationale for, and objectives of, the study clearly described?

Yes

Are sufficient details of the methods provided to allow replication by others?

Partly

Are the datasets clearly presented in a useable and accessible format?

Not applicable

Reviewer Expertise:

Osteoarthritis clinical research, epidemiology, health services research, implementation, evaluation, and outcomes research.

I confirm that I have read this submission and believe that I have an appropriate level of expertise to confirm that it is of an acceptable scientific standard.

# Peer review: Reviewer response for version 1

**DOI:** 10.21956/hrbopenres.14431.r29467

**Published:** 2021-05-24

**Authors:** Jocelyn L. Bowden

**Affiliations:** 1 Institute of Bone and Joint Research, the Kolling Institute, The University of Sydney, Sydney, NSW, Australia

Thank you for the opportunity to read your manuscript. You have presented the protocol for a scoping review that will identify and describe current research around the extent, nature and characteristics of different Models of Care delivering osteoarthritis care in primary care settings. I think this is a nicely written and very clear protocol that covers all the necessary components. It is timely and important contribution to the literature, especially given the growing focus on delivering evidence based care tailored to specific healthcare settings. I look forward to reading your results.

Is the study design appropriate for the research question?

Yes

Is the rationale for, and objectives of, the study clearly described?

Yes

Are sufficient details of the methods provided to allow replication by others?

Yes

Are the datasets clearly presented in a useable and accessible format?

Not applicable

Reviewer Expertise:

Osteoarthritis clinical research, models of care / service delivery, clinical trials, evidence based care

I confirm that I have read this submission and believe that I have an appropriate level of expertise to confirm that it is of an acceptable scientific standard.
